# Saving the Meniscus: A Retrospective Observational Study of the Incidence, Treatment, and Failure Rate of the Main Meniscal Tear Types at 24-Month Follow-Up

**DOI:** 10.3390/jcm14103350

**Published:** 2025-05-12

**Authors:** Daniele Screpis, Fjorela Qordja, Luca De Berardinis, Gianluca Piovan, Stefano Magnanelli, Andrea Amarossi, Antonio Pompilio Gigante, Claudio Zorzi

**Affiliations:** 1 Department of Orthopaedics, IRCCS Ospedale Sacro Cuore Don Calabria, 37024 Negrar di Valpolicella, Italy; daniele.screpis@sacrocuore.it (D.S.); piovan.gianluca@hotmail.it (G.P.); stefano.magnanelli@gmail.com (S.M.); andrea.amarossi@gmail.com (A.A.); claudio.zorzi@sacrocuore.it (C.Z.); 2 Clinical Orthopaedics, Department of Clinical and Molecular Science, School of Medicine, Università Politecnica delle Marche, Via Tronto, 10/a, 60126 Ancona, Italy; a.p.gigante@staff.univpm.it; 3 Orthopaedic Department, IRCCS Ospedale San Raffaele, Via Olgettina 60, 20132 Milan, Italy; luca8191deberardinis@gmail.com; 4 IRCCS INRCA Istituto Nazionale di Ricovero e Cura Per Anziani, Via S. Margherita, 5, 60124 Ancona, Italy

**Keywords:** suture, meniscus, horizontal cleavage tear, longitudinal tear, bucket-handle meniscal tear, radial tear, meniscal ramp lesions, meniscal root tear, complex meniscal tears, failure rate

## Abstract

**Background:** Despite advances in repair techniques, the failure rates of meniscal surgery are still high. The seven most common tear types—horizontal cleavage tears (HCTs), radial tears (RTs), meniscal ramp lesions (MRLs), meniscal root tears (MRTs), longitudinal tears (LTs), bucket-handle tears (BHMTs), and complex meniscal tears (CMTs)—were reviewed. The present retrospective observational study aimed to analyze their characteristics, incidence, treatment approach and failure rates of a consecutive cohort of patients undergoing meniscal arthroscopic repair. **Methods:** The database of a high-volume meniscal suture center was examined for lesions managed by all-inside, inside-out, outside-in, or transtibial pull-out techniques from January 2018 to September 2022. Demographic (gender, age at surgery, laterality of the affected knee) and intraoperative data (tear type/site, repair technique, and suture number/combination) were collected in order to calculate the failure rates of the cohort and of each tear type and suture technique. **Results:** Altogether, 636 procedures met our criteria of having at least a 2-year follow-up. The overall failure rate was 1.98%. The most frequent lesions were HCTs (41.98%), with most injuries being in the body/posterior horn (88.52%) of the right knee (56.92%). Treatment predominantly (92.50%) included all-inside sutures. All-inside repair had the highest failure rate (2.98%), followed by inside-out (1.56%) repair (*p* = 1.0), whereas outside-in and pull-out techniques never failed. Failure rates by lesion included BHMTs (7.27%), HCTs (2.25%), CMTs (1.49%), and LTs (1.25%); RMT, RML, and MRT repair were always successful. **Conclusions:** Findings at two years suggest that 1–3 all-inside sutures minimize MRL failure, whereas three or more all-inside sutures or combined techniques seem to be effective for HCTs, LTs, and RTs but not BHMTs. Pull-out repair worked best for complete tears/avulsion types of MRTs, whereas all-inside sutures effectively managed partial lesions. Results for CMTs were inconclusive.

## 1. Introduction

Meniscal injuries are common conditions accounting for 12% to 14% of all knee injuries, with an incidence of 60–70 cases per 100,000 people [1].

The menisci are primarily composed of type I collagen fibers within a highly hydrated extracellular matrix [2] and exhibit a zonal vascularization pattern (red-red, red-white, white-white zones), which critically influences their regenerative capacity and the outcomes of meniscal repair [3].

The menisci play a crucial role in knee joint homeostasis, preserving tibiofemoral congruency, joint stability, dynamic load distribution, and proprioception [4]. Notably, meniscal repair, by preserving meniscal tissue, restores biomechanical load distribution in the affected compartment, reducing the risk of early chondral wear and degeneration [5].

Treatment options for meniscal tears are both non-operative and surgical. The surgical options include meniscectomy and suture repair [1]. For several years, arthroscopic partial meniscectomy has been the most common orthopedic procedure in the world [6]. Recent studies suggest that patients treated with meniscectomy may have a substantially higher risk—estimated to be up to 300 times greater—of osteoarthritis progression and subsequent need for total knee arthroplasty compared to those managed with meniscal repair [7]. This growing body of evidence has contributed to a paradigm shift favoring meniscal preservation, often summarized by the principle ‘save the meniscus’ [8].

The most common meniscal tear types include horizontal cleavage tears (HCTs), radial tears (RTs), vertical tears, which encompass ramp lesions (MRLs), certain meniscal root tears (MRTs), and longitudinal tears (LTs), and complex meniscal tears (CMTs), which involve a combination of two or more types [1]. LTs may progress to bucket-handle meniscal tears (BHMTs).

HCTs split the meniscus into a superior and an inferior half, involving the articular surfaces and/or its periphery [9]. Their incidence is anywhere from 20 to 23% [10,11] to 40% [12] of all meniscal tears. RTs are meniscal tears arising from the central region (white-white) to the periphery (red-red) and can occur in all anteroposterior zones of the medial and lateral meniscus [13]. They induce increased joint contact pressure, involving a higher risk of chondral damage and meniscal extrusion than other tears. RTs have been reported to account for 10% to 23% of all tears in the adult population [14], 28% of medial meniscal tears [13], and 14% of isolated lateral meniscal tears [15]. LTs are vertical lesions running perpendicular to the tibial plateau and parallel to the long meniscal axis, among the circumferential collagen fibers [16]. They account for 18.2–37.6% [8] of all tears. BHMTs are large longitudinal vertical tears with an attached fragment that can be flipped into the intercondylar notch [17] and have a significant impact on knee biomechanics [18]. They account for nearly 10% [19] to 26% [20,21] of all meniscal tears. MRLs are vertical peripheral tears involving the posterior horn of the medial meniscus. They may lead to meniscocapsular or meniscotibial disruption [22]. Their true incidence is unknown due to high rates of mis- or underdiagnosis caused by low imaging sensitivity, poor intraoperative visualization, and surgeon experience [23]. MRLs are found in 9% [24] to 42% [22] of ACL reconstruction procedures, although isolated MRLs may exist in the absence of obvious ACL rupture [25]. An MRT is a meniscal lesion occurring within 1 cm of the meniscal root attachment, or a complete bony or soft tissue avulsion of the root attachment [26]. They account for up to 20% of all meniscal tears [27], although they have been labeled a “silent epidemic” due to their frequent underdiagnoses and the rapid progression of the untreated injuries into osteoarthritis [28]. Finally, CMTs involve multiplanar disruption of meniscal tissue [29] by a combination of two or more tear types [30]. Their incidence is around 15% of all meniscal injuries [31].

The indications and techniques for surgical repair depend on multiple patient- and lesion-related characteristics [32]. The type of meniscal tear is critical in guiding treatment decisions [33]. Second-generation all-inside, inside-out, outside-in [32], and transtibial pull-out sutures are the most widely used techniques, optimizing the benefits of the surgical treatment and minimizing its drawbacks. Yet, despite advances in repair techniques and devices, failure rates still range from 10% [1] to 36% [8].

Currently, the literature includes numerous studies that analyze individual meniscal lesions in detail, focusing on incidence, classification, diagnosis, treatment, outcomes, and failure rates. Similarly, several works provide a general overview encompassing the most common meniscal injuries; however, these often lack specific and clinically relevant details for each lesion. To date, a descriptive study addressing the most frequent meniscal tears, highlighting intraoperative findings and short-term treatment evolution, is still missing.

We describe meniscal repair as it occurred in our high-volume meniscal suture center and analyze the most common meniscal lesions with emphasis on their features and treatment. The primary aim of the study is to assess the frequency, site (medial/lateral), radial location (anterior horn, body/posterior horn, root), and surgical management of each type. The secondary aim is to analyze total and lesion-specific failure rates with a focus on the suture techniques related to higher failure rates. In light of the current literature and of lesion characteristics, we hypothesize that failure rates differ across lesions and that certain repair patterns may be at a higher risk of failure than others.

## 2. Materials and Methods

### 2.1. Study Design and Patient Selection

The present retrospective observational study is on a consecutive cohort of patients undergoing meniscal repair.

Following review board approval, the institutional electronic database was mined for the meniscal arthroscopic repair procedures performed by the same senior surgeon from January 2018 to September 2022 at the Orthopedic Department of IRCCS Sacro Cuore-Don Calabria Hospital (Negrar di Valpolicella, Italy), reference center for orthopedic surgery, with a high annual volume of procedures in the field of sports medicine.

### 2.2. Inclusion and Exclusion Criteria

Procedures involving the seven most common types of meniscal lesions—HCTs, RTs, LTs, BHMTs, MRLs, MRTs, and CMTs—that were treated with all-inside, outside-in, inside-out, and transtibial pull-out suture techniques were included. In particular, only patients who had a follow-up of at least 2 years were selected. The study excluded lesions treated solely with regularization; lesions requiring treatments other than those listed above (e.g., meniscal transplantation or anchor-based sutures) or additional biological treatments; cases with less than 2-year follow-up; patients with prior knee infections or discoid meniscus; other less frequent meniscal lesion types; and any procedures whose description in the surgical records was unclear. Finally, patients with confounders such as comorbidities that potentially impair meniscal healing following repair procedures (rheumatic diseases, tumors, or coagulative disorders) or chondral damage >2 according to the International Cartilage Repair Society classification were excluded from the study.

### 2.3. Minimizing Bias

Several strategies were employed to minimize bias in this retrospective study, particularly considering the non-randomized nature of treatment allocation due to the distinct types of meniscal lesions. Despite the lack of randomization, treatments were systematically selected based on the type and location of the lesion, which could help reduce selection bias that may influence the results. Data were collected using a standardized protocol for all cases, and all patients were evaluated using the same criteria to determine meniscal repair failure. The patient cohort included a wide range of characteristics, both in terms of meniscal type and surgical treatment. Additionally, diagnostic criteria for each type of lesion and the definition of failure were clearly defined in advance to minimize discrepancies between clinicians. To further reduce information bias, a double data review was conducted: an independent evaluation by two authors (F.Q. and L.D.B.), with any ambiguous cases resolved by a third, experienced author (D.S.). Finally, all surgeries were performed by a single surgeon, which helped minimize inter-surgeon bias that could affect the outcomes.

### 2.4. Data Collection

We abstracted the following demographic and preoperative data from the dataset: gender, age at surgery, and affected knee (left/right). We recorded the following intraoperative data: tear type, based on the intraoperative arthroscopic findings, instead of the preoperative diagnosis; tear laterality (medial/lateral); tear site (anterior horn, body/posterior horn, root); repair technique (all-inside, inside-out, outside-in, pull-out); number and combination of sutures used; and presence of a meniscal lesion in the contralateral meniscus.

We also recorded treatment failures, defined as the need for further surgery in case of tear persistence [34], to calculate the overall failure rate of the cohort and of each type of tear and repair technique.

### 2.5. Tear Types: Classification and Diagnosis

#### 2.5.1. Horizontal Cleavage Tears

HCTs are graded on a 1 to 3 scale based on magnetic resonance imaging (MRI) scans according to Stoller et al. [35]. In this classification, grade 1 involves irregularly marginated intrameniscal signal; grade 2 involves primarily linear signal that does not communicate with an articular margin; and grade 3 involves signal intensity that communicates with an articular margin. On MRI, HCTs usually appear as an area where signal alteration extends horizontally along the meniscus parallel to the tibial plateau.

#### 2.5.2. Radial Lesions

The latest RTs classification, proposed by Chahla et al. [5] and adopted in this study, divides them into partial stable RTs extending to the white-white zone (type I); partial unstable RTs extending to the red-white zone (type II); complete RTs without gapping (type III), complete RTs with 3 mm gapping (type IV), and complete RTs with >3 mm gapping (type V). We adopted the MRI-based criteria described by Harper et al. [36], where on MRI scans the posterior and anterior meniscal horns appear as low-signal triangles on sagittal scans. In contrast, the body has a triangular shape on coronal images. All RTs shared the truncated triangle sign (abrupt termination of the triangular meniscal contour at its tip on sagittal or coronal images) and the cleft sign (linear, vertical high signal extending through the meniscus on coronal or sagittal images). They also shared the marching cleft sign, where a cleft running in central or peripheral direction is detected on consecutive sagittal or coronal images.

#### 2.5.3. Longitudinal Lesions

There is no universally accepted classification for LTs. Two widely used systems are based on circumferential zones [37] or meniscal radial zones [4]. Circumferential zones consist of zone 0, the meniscosynovial junction; zone 1, the outer third; zone 2, the middle third; and zone 3, the inner third. Radial zones categorize LTs based on their extension into the posterior, midbody, or anterior zones. MRI findings for LTs [38] are linear hyperintensity on T2-weighted images; disruption of meniscal morphology with alterations running parallel to the circumferential fibers; meniscal extrusion (≥3 mm beyond the tibial plateau); and associated joint effusion.

#### 2.5.4. Bucket-Handle Meniscal Tears

BHMTs can be classified according to Lim et al. [39] based on reducibility and tear type into 3 types: type 1 rotates upward, type 2 rotates downward (both are easily reduced), whereas in type 3 the displaced central fragment is rotated by 180° or more and is difficult to reduce. MRI scans are evaluated in the sagittal plane for subchondral edema, the double posterior cruciate ligament sign [20], the flipped meniscus sign, the absent bow sign, the disproportionate posterior horn sign, and/or the double anterior horn sign [40]. In the coronal plane, they are examined for the intercondylar notch sign [41] and in the axial plane for the V-sign and/or the double anterior cruciate ligament (ACL) sign [40].

#### 2.5.5. Meniscal Ramp Lesions

The most recent MRL classification, and the one adopted herein, is by Pimprikar and Patil [42]. Type 1 involves injury only to the meniscocapsular junction without meniscotibial ligament (MTL) disruption and may only affect the synovium. Type 2 involves the meniscocapsular ligament (MCL) and the MTL, causing the posteromedial capsule to peel off from the inferior surface of the meniscus. Type 3 involves meniscal tissue, MCL, and MTL. MRLs can be detected on routine MRI. The most obvious finding is an irregularity or a longitudinal vertical tear in the peripheral zone of the posterior horn of the medial meniscus, with interposed thin fluid signal between the posterior horn of the medial meniscus and the posteromedial capsule [43]. Additionally, there may be soft tissue edema between the meniscus and the collateral ligament, bone bruising in the posteromedial tibia from pivot shift, countercoup injury in the medial compartment, and anterior translation of the medial plateau in relation to the femoral condyle [22].

#### 2.5.6. Meniscal Root Tears

We have adopted the classification of LaPrade et al. [44], which divides tears into five categories based on location and quality. Type 1: isolated, partial, and, therefore, stable root tears; type 2: complete root tear; type 3: tear with a concomitant longitudinal/circumferential tear (i.e., BHMT); type 4: oblique meniscal tear extending into the root, resulting in complete root detachment; type 5: complete bony avulsion fracture of the root from its attachment. Unlike other classifications, the LaPrade system applies to both the anterior and posterior roots [28]. MRI is the most reliable non-invasive modality for MRT diagnosis [28]. Specific signs include increased signal due to fluid collection near the roots, the ghost sign (sagittal cuts where the meniscus is not identifiable in its normal position), and meniscal extrusion >3 mm from the tibial articular cartilage in coronal views. A further sign is subchondral insufficiency fracture of the knee, i.e., bony edema of the femoral condyles or tibial plateau that seems to be caused by increased point-loading due to lack of meniscal support [26].

#### 2.5.7. Complex Meniscal Tears

There is currently no classification for CMTs. MRI is the diagnostic gold standard, the signal being dependent on the imaging patterns of each simple lesion within the CMT.

### 2.6. Types of Repair

The main exposure in this study refers to the type of meniscal repair technique employed, specifically the *all-inside*, *inside-out*, *outside-in*, and *pull-out*. The suture techniques employed to repair meniscal tears are based on lesion type and location. The four most widely used approaches are also those applied in the cases reviewed here.

#### 2.6.1. All-Inside Technique

All-inside repair was performed through standard arthroscopic portals using the FAST-FIX^TM^ Meniscal Repair System (Smith & Nephew, Andover, MD, USA) according to the manufacturer’s recommendations. Briefly, each FAST-FIX contains two 5 mm polymer-integrated bio-inert anchors (PEEK-OPTIMA^®^ from Invibio^®^, Lancashire, UK) with a pretied, self-sliding knot comprising a 2-0, non-absorbable, UHMW polyethylene ULTRABRAID^TM^ suture. The entire system is packaged in an easy-to-insert integrated delivery needle. Sutures were placed until the desired stability was achieved.

#### 2.6.2. Inside-Out Technique

A long and straight needle loaded with 2-0 non-absorbable sutures is inserted through an arthroscopic cannula and passed from the intra-articular side through the meniscus toward the outer capsule using a dedicated inside-out meniscal repair device. Sutures are placed approximately 1–2 mm from the tear margin to ensure optimal stabilization. To prevent iatrogenic injury, a small incision is made on the medial or lateral aspect of the knee at the level of the meniscus, and a spoon-shaped retractor is introduced to shield neurovascular structures. Once the sutures are successfully passed, both ends are retrieved through the extra-articular incision, where they are securely tied under direct visualization, ensuring adequate compression of the meniscal tissue while avoiding excessive tension that could compromise vascular supply.

#### 2.6.3. Outside-In Technique

The outside-in technique involved using a 22G sterile lumbar spinal needle and slowly absorbable suture material (PDS 0). To prevent cheese-wiring, the second suture was crossed over the first.

#### 2.6.4. Transtibial Pull-Out Technique

The transtibial tunnel pull-out technique is principally used for MRTs. A 3 cm incision is made adjacent to the tibial tubercle on the side of the tear. A tibial tunnel guide, opened at 60°, is used to ream the tunnel. Anatomical positioning of the tunnels in the posterior root attachment site should be visualized arthroscopically. Two simple sutures are passed through the meniscal root and shuttled down the tibial tunnel. After root reduction, the sutures through the tibial tunnel are tied over a button on the anterior tibia to avoid suture cut-in [28].

### 2.7. Surgical Procedure

The patient in supine position was examined under anesthesia. The limb was placed in standard arthroscopic position with padding of all bony prominences. Following spinal anesthesia, tourniquet placement, and a time-out, standard medial and lateral transpatellar portals were opened, and diagnostic arthroscopy was performed to confirm the preoperative diagnosis. Once it was established that the tear was amenable to repair, its edges were freshened up using a shaver. One of the four repair techniques described above was then applied depending on lesion type and location. Regardless of repair type, sutures were placed along the tear at 5–7 mm intervals [45,46] until achievement of stability to probing. The knee was then copiously irrigated and closed in standard layered fashion.

### 2.8. Postoperative Rehabilitation

All patients followed the same rehabilitation protocol, designed to maximize healing and functional recovery and to eliminate an important potential confounder. Weight bearing was not allowed for three weeks, followed by progressive partial load for the next two weeks. Crutches must be used for six weeks after surgery. Patients wore a brace locked in extension for the first ten days, unlocking to 0–60° in the following week, and to 0–90° in the subsequent week. Once adequate quadriceps strength was achieved, typically around four to five weeks, the immobilizer brace was discontinued, with a progressive increase in flexion. Initially, exercises focused on quadriceps sets, straight-leg raises, heel slides, and patellar mobilization. Subsequently, closed-chain exercises, lunges (0–90°), leg presses (0–90°), proprioceptive training, and a stationary bike were gradually introduced. From weeks 12 to 16, patients progressed to further strengthening exercises, including single-leg strengthening, jogging, and a gradual transition to running. Sport-specific exercises were incorporated as recovery progressed.

### 2.9. Statistical Analysis

We used Microsoft Excel (version 16.75.2, Redmond, WA, USA) in conjunction with the XLSTAT resource pack (XLSTAT-Premium, Addinsoft Inc., New York, NY, USA) for all analyses. All continuous data are expressed as mean and standard deviation (SD), whereas categorical variables are expressed as frequency and percentages. The Chi-square test or Fisher’s exact test was used for categorical data as appropriate. A *p*-value < 0.05 was statistically significant.

## 3. Results

**Figure 1:**
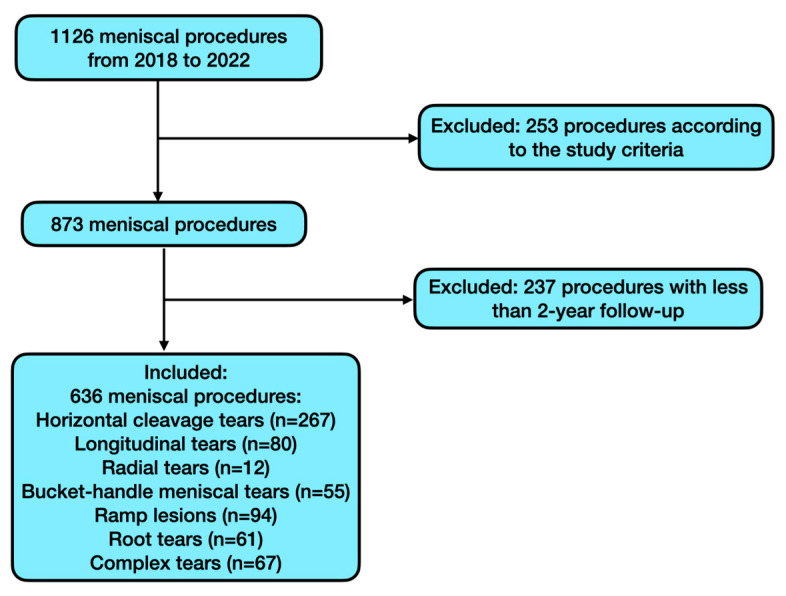
Patient selection flow chart.

The patient selection flow chart after application of the inclusion and exclusion criteria is shown in Figure 1 below.

The search of the institutional database yielded 1126 meniscal repair procedures performed by the same senior surgeon between January 2018 and September 2022. A total number of 636 procedures (518 patients) met the study criteria. The mean patient age was 30.69 years (SD, 12.94). Of the patients, 349 (67.37%) were men and 169 (32.63%) were women. The average BMI was 23.6 (SD, 2.60).

Altogether, 274 (43.08%) procedures involved the left knee and 362 (56.92%) the right knee. The mean follow-up was 51 months (SD, 16.56). The overall failure rate was 1.89% (12/636).

### 3.1. Overview of General Results

Table 1 provides an overview of tears and patient data. The body/posterior horn accounted for 563 cases (88.52%), the meniscal roots for 61 cases (9.59%), and the anterior horn for 12 cases (1.89%). The most common lesion type were HCTs (n = 267, 41.98%), followed by MRLs (n = 94, 14.78%) and LTs (n = 80, 12.58%), whereas CMTs (n = 67, 10.53%), MRTs (n = 61, 9.59%), and BHMTs (n = 55, 8.65%) had similar frequencies. The least numerous lesions were RTs (n = 12, 1.89%). Of the 1160 sutures, 1073 (92.50%) were performed with the all-inside technique, 64 (5.52%) with the inside-out technique, 12 (1.03%) with the transtibial pull-out technique, and 11 (0.95%) with the outside-in technique. Of the 636 procedures, 12 (1.89%) failed. The failure rate was higher for the medial (10/359; 83.33%) than the lateral meniscus (2/277; 16.67%), with a *p*-value of 0.08. As for the applied techniques, all-inside recorded the highest failure rate with 32 failures out of 1073 cases (2.98%), followed by the inside-out (one failure out of 64 cases, 1.56%). No failures were observed for the outside-in or pull-out techniques. Fisher’s exact test yielded *p* = 1.0, indicating that there is no statistically significant difference in failure rates among the suture techniques.

### 3.2. Horizontal Cleavage Tears

We identified 267 HCTs, predominantly (95.88%) affecting the body/posterior horn of the medial meniscus (55.80%). However, the HCT’s distribution between the medial and lateral meniscus does not reach statistical significance (*p* = 0.058). Altogether, they accounted for 405 sutures, predominantly (94.32%) using the all-inside technique; in 58.43% of these cases, it was a single all-inside suture. All six (2.25%) failures affected the medial meniscus with a statistical difference compared to the lateral one (*p* = 0.036); five (83.33%) of these cases involved one or two all-inside sutures. Repair with three sutures involved only one failure, whereas a higher number or a combination of different sutures never yielded complications. Details about HCTs are provided in Table 2.

### 3.3. Radial Tears

The search yielded 12 RLs (Table 3), predominantly (91.67%) involving the body/posterior horn of the lateral meniscus (75%). They were treated with 31 meniscal procedures: the all-inside technique was used in 77.42% of cases, most commonly (33.33%) with two all-inside sutures. There were no failures.

### 3.4. Longitudinal Lesions

There were 80 LTs, all affecting the body/posterior horn and mostly the lateral meniscus (51.25%). Their management required 154 procedures, nearly all performed with the all-inside technique (97.40%), mostly with two sutures. There was only one (1.25%) failure, involving a single all-inside suture, as shown in Table 4.

### 3.5. Bucket-Handle Meniscal Tears

All 55 BHMTs involved the body/posterior horn and were more numerous (61.82%) in the medial meniscus. Treatment involved 193 procedures, most commonly (85.49%) using the all-inside technique with a mean of 3.5 sutures/lesion. The failure rate was 7.28%, with three cases involving the medial and one case involving the lateral meniscus, all treated with three or more sutures (Table 5).

### 3.6. Meniscal Ramp Lesions

We identified 94 MRLs, all treated with the all-inside technique. A single suture was used in 55 (58.51%) cases. There were no failures, as depicted in Table 6.

### 3.7. Meniscal Root Tears

Table 7 shows that there were 61 MRTs, primarily (86.89%) affecting the lateral meniscus. LaPrade type I lesions were the most prevalent (80.33%). Repair required 82 meniscal procedures, most often (85.37%) with the all-inside technique and with single all-inside suture (55.74%). There were no failures.

### 3.8. Complex Meniscal Tears

The search yielded 67 CMTs, all affecting the body/posterior horn and predominantly the lateral meniscus (52.24%). Table 8 shows that there were 159 repairs, almost exclusively (91.82%) with the all-inside technique and most often (29.85%) with two all-inside sutures. The only failure involved three all-inside sutures performed in the medial meniscus.

## 4. Discussion

This study offers an overview of the incidence of the main types of meniscal injuries and repair procedures and reports their failure rates.

### 4.1. Failure Rates

There is no consensus on the optimal management approach for the main types of meniscal lesions [13,47,48,49,50,51]. The failure rate of our 636 procedures, performed by a single senior surgeon at a high-volume center, at the two-year follow-up was 1.89%. This rate is considerably lower than those reported in most studies, which range from 9.4 to 15% [1,6,52,53] to up to 36% [8,34,54]. In addition, although we found a higher failure rate (1.57%) for the medial than the lateral (0.31%) meniscus, the difference was not significant. This finding contrasts with recent data by Schweizer et al. [52], who described a significantly lower failure rate for lateral than medial meniscal repair (6.1% and 10.8%, respectively).

### 4.2. Suture Techniques

Given the inconclusive clinical data regarding the most appropriate suture technique for each tear type, surgeon preference usually prevails, and indeed our data do not show a clear superiority of any one approach [48]. As suggested by Saltzman et al. [20], surgeons likely apply the technique with which they are most comfortable.

It is important to consider the advantages and disadvantages of each technique. In fact, inside-out repair [48] seems to remain the gold standard of meniscal repair due to its success in a variety of meniscal zones, lower cost, and ability to deploy multiple sutures with minimal damage to the meniscus [13]. Drawbacks [55] include an extra assistant for passing sutures and an extra incision [48], a certain risk of complications, longer surgical time, and postoperative pain. Second-generation all-inside devices, which leave a single suture inside the joint while the PEEK pledgets remain outside the capsule, reduce operative time and have potentially lower complication rates. They have proved to be biomechanically similar to inside-out suture techniques. Drawbacks include the increased up-front expense, the possibility of device breakage or misfire, and the potential for vascular injury when tears involve the posterior horn [55]. The outside-in technique can be useful for anterior tears that are difficult to reach through arthroscopic portals [48]. Its benefits include small incisions, low neurovascular risk, and a minimally invasive approach that avoids leaving prominent intra-articular material [56]. Transtibial tunnel root repair seems to be superior to other techniques in terms of anatomical restoration of joint kinematics, including contact pressures and hoop stress [28]. Potential limitations include the use of transtibial tunnels, which may cause concern for tunnel convergence or interference in patients needing multiple concomitant procedures [28].

In our study, the approach with the highest absolute failure rate was the all-inside technique (2.98%, 32/1073), followed by inside-out (1.56%, 1/64) repair, without statistical significance (*p* = 1.0). Outside-in and pull-out repairs never failed. Fillingham et al. [57] described comparable functional outcomes and similar anatomical and clinical failure rates (respectively, 11% and 10%) for inside-out repair and modern all-inside approaches. Doral et al. [32] also found similar complication rates with the two techniques. Okezi et al. [4] presented similar loads to failure in biomechanical tests with both techniques. In contrast, Borque et al. [34] reported that in a cohort of elite athletes, the all-inside repair of medial tears involved a higher failure rate than inside-out repair of medial or lateral tears. A recent meta-analysis by Schweizer et al. [52], involving 3921 menisci from 51 studies, found no significant differences in the pooled failure rates of all-inside and inside-out approaches.

### 4.3. Horizontal Cleavage Tears

HCTs were the most frequent tear type in our population, accounting for almost 42% of lesions. They more often affected the medial (55.80%) than the lateral (44.20%) meniscus, *p* = 0.058. Our data are in line with a systematic review [4] that found no significant differences in the number of HCTs involving the lateral (42.5%) and medial (57.5%) meniscus. The finding that 95.88% of HCTs affected the body/posterior horn and only 4.12% involved the anterior horn also agrees with the review’s data. Regarding technique, all-inside sutures were the most common (94.32%), most often (58.43%) with a single all-inside suture. This agrees with the report by Morris et al. [12] that the technique applied most frequently was the all-inside approach (42%), followed by inside-out (7%) and outside-in (4.1%) repair. The HTC failure rate was 2.25%, exclusively involving the medial meniscus, most often the body/posterior horn. A statistically significant difference was observed regarding failure rates between the medial and lateral meniscus (*p* = 0.036). Most failures (83.33%) regarded cases with one or two all-inside sutures. There was only one failure with three sutures, whereas a larger suture number or a combination of techniques never yielded complications. These data disagree with most of the literature, as only Sallé de Chou et al. [10] reported comparable failure rates (3.7% up to 10 years), albeit in a cohort of 10 patients treated by open suture repair. In contrast, Morris et al. [12] described an overall reoperation rate of 11.4% in 289 HCTs. Slightly higher reoperation rates, of 15.3% [58] and 17.3% [59], respectively, have been reported in similar-sized cohorts of children and adolescents. Lee et al. [51] found a failure rate of 39% in 36 isolated HCTs treated surgically with a minimum of 2-year follow-up; however, they did not provide treatment details. All this considered, HCTs seem difficult to manage due to the risk of vascular damage [60], narrow residual volume, and consequent increase in peak contact pressure [9]. Their treatment is debated [51]. Analysis of our data highlighted that three or more all-inside sutures or a combination of all-inside and other suture techniques yielded a failure rate close to zero.

### 4.4. Meniscal Ramp Lesions

MRLs (94/14.78%) were the second most common type. All MRLs were treated with the all-inside technique using a single all-inside suture in 58.51% of cases. Alessio-Mazzola et al. found good clinical results and low complication rates regardless of the technique used [61]. Similarly, according to a systematic review [50], there is no gold standard technique for MRLs, and the surgeon should be familiar with the different repair options, a view that mirrors the conclusions of Siboni et al. [46]. In contrast, a systematic review and meta-analysis by Marin et al. [62] highlighted an association between suture techniques and outcomes based on second-look arthroscopy, as the authors found complete healing predominantly (82.1%) in patients managed by all-inside repair, whereas incomplete healing (15.4%) was more common with inside-out sutures. A biomechanical study of all-inside sutures passed through standard anterior portals in cadavers demonstrated that they provide reliable fixation [63]. According to some critics, the all-inside technique is at significantly higher risk of failure than suture hook repair [64]. Considering the outcomes, MRL repair was invariably successful, whereas most reports describe failure rates of 2.6–12.0% [61,64,65,66]. Our data agree with those of Karaca et al. [45], who found 0/41 in isolated MRLs treated with all-inside sutures through standard portals, even though the sample they analyzed was nearly half the size of our cohort. Similarly, in a study using the same second-generation all-inside device as ours [67], control arthroscopy showed complete healing in 40/46 patients, incomplete healing in five, and failure in one. In conclusion, MRLs are frequent and must be repaired to prevent their possible evolution to medial BHMTs [68]. Treatment with the appropriate number of all-inside sutures according to the anteroposterior extension of the tear [69] appears to ensure complete success.

### 4.5. Longitudinal Tears

LTs were the third most common type, accounting for 12.58% of cases. All 80 tears affected the body/posterior horn, and 51.25% involved the lateral meniscus. LT management required 154 sutures, nearly all (97.40%) performed with the all-inside technique, mostly (50%) with two all-inside sutures. According to Kurnaz et al. [70], LTs can be repaired by all-inside, inside-out, and outside-in approaches, although stitch configuration proved to be more important to repair stability than the approach used. A systematic review of human cadaveric studies [71] identified a variety of techniques suitable for LT repair and listed their pros and cons. In a retrospective study of LTs treated with the FAST-FIX 360° Meniscal Repair System^®^, Grossi et al. [72] found no failure or major complications and suggested that the all-inside technique is safe and effective for LTs. A systematic review of LT treatment in terms of contact pressure/area and tensile properties [73] identified no significant differences for the first parameter among all-inside, inside-out, and outside-in techniques. For the second parameter, results were inconclusive. As for the only failure (1/80; 1.25%), it concerned a single all-inside suture. Ouanezar et al. [74] described comparable outcomes in 200 patients managed with the all-inside technique. The failure rate at 45 months was 3.5%. In contrast, Uzun et al. [75] found a rate of 11.6% in 43 repairs using inside-out, all-inside, or a combination thereof at a mean interval of 63 months. Unfortunately, both studies included only lateral meniscal LTs. According to Noyes et al. [76], only 62% of 33 repairs succeeded at a mean follow-up of 11 years. Recently, Zicaro et al. [77] described a failure rate of 6.6% in 119 patients with isolated LTs at a mean follow-up of 7 years, without differences between the menisci. Altogether, LTs most commonly affect the body/posterior horn of the lateral meniscus. Treatment with two or more all-inside sutures resulted in a failure rate close to zero.

### 4.6. Complex Meniscal Tears

CMTs accounted for approximately 10% of injuries and always involved the body/posterior horn, more frequently (52.24%) of the lateral meniscus. Ahmed et al. [31] found that they ranked second after BHMTs. For this type of lesion, repair was almost exclusively (91.82%) performed with the all-inside technique, mostly with two all-inside sutures (29.85%). CMTs, particularly those involving the poorly vascularized white zone, used to be considered irreparable, owing to their low healing potential, and were managed by meniscectomy [78]. More recent studies show that repair yields better functional outcomes and quality of life [79,80,81]. As CMTs often involve several segments of the meniscus, inside-out, inside-out, and/or all-inside repair may be required to ensure optimal outcomes [4]. We found a single failure, affecting the medial meniscus. Treatment involved three all-inside sutures. However, the failure of sutured CMTs is unpredictable, both due to their intrinsic heterogeneity—as each simple lesion evolves in a different way—and to the very limited literature [78]. A meta-analysis of studies that predominantly included CMTs [54] found an overall failure rate of 19.1% after meniscal repair with non-augmented repair techniques at 7.1 years. Another meta-analysis by Nepple et al. [82], including 27 studies with a minimum follow-up of five years, demonstrated an overall failure rate of 22.6% and a pooled modern device failure rate of 19.5%. Recently, Vicens et al. [78] reported a total failure rate of 9.5% at a mean follow-up of 33 months for CMTs managed by a meniscal wrapping technique. Unfortunately, the literature on CMTs is limited. The heterogeneous tear combination, location, and healing potential prevent drawing conclusions regarding treatment and may involve challenges to the surgeon [29].

### 4.7. Meniscal Root Tears

MRTs were the fifth most common type of meniscal lesions (9.59%) and primarily affected the lateral meniscus (86.89%). LaPrade I tears were the most frequent (80.33%). In the literature, they account for 10–21% of all tears [83], medial posterior MRTs being more common (52%) than lateral posterior MRTs (41%) [84]. Altogether, there were 82 procedures (85.37% all-inside sutures). The most common treatment involved a single all-inside suture (55.74%). Treatment can differ slightly depending on the type of root tear [85]. According to Simonetta et al. [65], MRTs can be managed by side-to-side reconstruction or transtibial pull-out repair, according to subtype. In line with Zheng et al. [86], side-to-side suture is successful in radial tears with an adequate root remnant, whereas pull-out repair yields better results in patients with root avulsion or radial tears with an inadequate meniscal remnant. As for failure, MRT repair was always successful in our cohort. Transtibial repair of these tears has a low failure rate, with an observed revision rate of 6.7% [26]. As regards side-to-side repair, complete or partial healing has been reported in 93.6% of second-look arthroscopies [86]. Patients undergoing lateral meniscal root repair may benefit slightly more than those undergoing medial meniscal root repair [87]. To sum up, repair of MRTs, which are most frequently located posteriorly in the lateral meniscus, is often successful. The findings reported above suggest using all-inside repair for LaPrade I lesions and transtibial pull-out repair for LaPrade II-V lesions.

### 4.8. Bucket-Handle Meniscal Tears

BHMTs were the second least common type (55/8.65%). They were exclusively located in the body/posterior horn, with a higher prevalence in the medial meniscus (61.82%). The literature reports a 1.67% incidence of isolated BHMTs [21]. Repair involved a mean of 3.5 sutures/lesion, most commonly by the all-inside technique (85.49%). According to Samuelsen et al. [55], the most common BHMT suture techniques are inside-out and all-inside, the former having long been considered the gold standard. Indeed, biomechanical studies support the increased use of all-inside devices, demonstrating similar loads to failure when comparing second-generation all-inside suture devices with vertical mattress suture configurations. These findings agree with the meta-regression analysis by Costa et al. [88], who failed to identify significant differences between all-inside and inside-out repair. A combination of all-inside and inside-out techniques has been suggested to improve the mechanical strength of the repair [48]. Among all tears, BHMTs had the worst outcomes, with a failure rate of 7.28%, primarily (75%) affecting the medial meniscus. It is reported that most (92.3%) failures seem to occur within two years [88]. According to Saltzman et al. [20], failure does not appear to be associated with repair technique or suture number. BHMTs have worse clinical outcomes than smaller vertical LTs [48]. Fillingham et al. [57] found slightly higher rates, namely 11% for all-inside and 10% for inside-out repair, with a mean of two and three sutures, respectively. Samuelsen et al. [55] reported an overall failure rate of 20% for all-inside and inside-out techniques. Similarly, El Helou and colleagues found that all-inside repair was the most common (45.1%) technique for medial BHMTs (failure rate, 20.2%), and that it was more than four times more likely to fail than a combination of techniques. Finally, Ardizzone et al. [48] reported a failure rate of all-inside repair of 29.3% at an average of 13.0 months, without differences between medial and lateral menisci. In summary, BHMTs are difficult to treat. A higher suture number and/or combination of techniques increased the failure rate, probably due to tissue trauma.

### 4.9. Radial Tears

RTs were the least common, with only 12/636 (1.89%) cases. Most (66.67%) were type II lesions according to Chahla and colleagues, and 75% involved the lateral meniscus. In the literature, RTs account for up to 28% of medial meniscal tears [13]. The 12 lesions required 31 sutures, predominantly (77.42%) with the all-inside technique; two all-inside sutures were the most common pattern (33.33%). They were managed as suggested by Chahla et al. [5]. Accordingly, type II lesions were treated with all-inside mattress sutures and type III lesions with a combination of all-inside and out-in techniques, with a predominance of all-inside, whereas type IV lesions were primarily treated with inside-out and outside-in techniques, sometimes combined with all-inside repair (type I lesions were excluded as they were debrided, and there were no type V tears). There is no consensus on the best repair technique for RTs. According to a systematic review [89], all-inside and inside-out sutures yielded improved functional outcome scores; however, inside-out repair was associated with higher clinical failure rates and lower healing rates on second-look arthroscopy than all-inside repair. Mameri et al. favor all-inside repair [13]. Radial lesion repair never failed, in contrast with the literature. A recent systematic review reported an overall failure rate of 8.0% [90], whereas 13% has been described in the lateral meniscus [15]. As there were only 12 RTs, it is impossible to draw firm conclusions. However, the treatment suggested by Chahla and colleagues [5] resulted in no failures.

### 4.10. Limitations

First of all, this is a retrospective study. Secondly, this study was conducted in a high-volume surgical center by an expert surgeon, potentially influencing the outcomes and limiting the generalizability of the results to other centers. However, avoiding multiple surgeons could potentially reduce bias. Thirdly, the relatively short-term follow-up of two years limits the ability to predict long-term failure rates. Furthermore, the study lacks clinical analysis of patients’ outcomes or activity levels. Additionally, the low incidence of some lesions prevented drawing firm conclusions. Moreover, the unpredictable clinical behavior of other lesions in terms of pattern and treatment approach further limited the ability to reach conclusions. In addition, the current study is missing sensitivity or subgroup analyses. Finally, as control MRI or second-look arthroscopy were not performed routinely even in those asymptomatic patients, failure rates may have been underestimated.

## 5. Conclusions

A larger number of sutures (>three all-inside) or combinations of suture techniques effectively reduced failure rates in HCTs and LTs. On the contrary, the use of more than three sutures appeared associated with a higher rate of failure for BHMTs. MRLs treated with 1 to 3 all-inside sutures never failed, although this depended on lesion extent. LaPrade I MRTs, which were reattached with pull-out sutures, and LaPrade II-V tears. For RTs, successful outcomes involved a combination of all-inside and inside-out techniques. Finally, no firm conclusions could be drawn for CMTs. Future prospective studies are therefore necessary to validate treatment approaches for the most common meniscal injuries, aiming to minimize the risk of failure.

## Figures and Tables

**Table 1:** Overview of general results.

Variables	Both Menisci	Medial Meniscus	Lateral Meniscus
	Number (%)	Number (%)	Number (%)
Patients	518	343 (66.22)	175 (33.78)
No. of procedures	636	359 (56.45)	277 (43.55)
Radial location			
Anterior horn	12 (1.89)	0 (0)	12 (100)
Body/posterior horn	563 (88.52)	351 (62.34)	212 (37.66)
Meniscal root tears	61 (9.59)	8 (13.11)	53 (86.89)
Tear type			
Horizontal cleavage tears	267 (41.98)	149 (55.81)	118 (44.19)
Meniscal ramp lesions	94 (14.78)	94 (100)	
Meniscal root tears	61 (9.59)	8 (13.11)	53 (86.89)
LaPrade I	49 (80.33)	7 (14.29)	42 (85.71)
LaPrade II–V	12 (19.67)	1 (8.33)	11 (91.67)
Longitudinal tears	80 (12.58)	39 (48.75)	41 (51.25)
Complex meniscal tears	67 (10.53)	32 (47.76)	35 (52.24)
Bucket-handle tears	55 (8.65)	34 (61.82)	21 (38.18)
Radial tears	12 (1.89)	3 (25)	9 (75)
Chahla II	8 (66.67)	3 (37.5)	5 (62.5)
Chahla III	2 (16.67)	0 (0)	2 (100)
Chahla IV	2 (16.67)	0 (0)	2 (100)
Chahla V	0 (0)	0 (0)	0 (0)
No. of repairs	1160	634 (54.66)	526 (45.34)
All-inside	1073 (92.50)	613 (57.13)	460 (42.87)
Inside-out	64 (5.52)	19 (29.69)	45 (70.31)
Outside-in	11 (0.95)	1 (9.09)	10 (90.91)
Pull-out	12 (1.03)	1 (8.33)	11 (91.67)
Overall failure rate	12 (1.89)	10 (83.33)	2 (16.67)

**Table 2:** Horizontal cleavage tears.

Variables	Both Menisci		Medial Meniscus		Lateral Meniscus	
	Number (%)	Failures (%)	Number (%)	Failures (%)	Number (%)	Failures (%)
No. of tears	267	6 (2.25)	149 (55.80)	6 (4.03)	118 (44.20)	0 (0)
Radial location						
Anterior horn	11 (4.12)		0 (0)		11 (9.32)	
Body/posterior horn	256 (95.88)		149 (100)		107 (90.68)	
No. of repairs	405		225 (55.56)		180 (44.44)	
AI	382 (94.32)		225 (100)		157 (87.22)	
IO	20 (4.94)		0 (0)		20 (11.11)	
OI	3 (0.74)		0 (0)		3 (1.67)	
Technique(s) applied						
1 AI	156 (58.43)	2 (33.33)	83 (55.70)	2 (33.33)	73 (61.86)	0 (0)
2 AI	79 (29.59)	3 (50)	56 (37.58)	3 (50)	23 (19.49)	0 (0)
3 AI	16 (5.99)	1 (16.67)	10 (6.71)	1 (16.67)	6 (5.08)	0 (0)
4 AI	2 (0.75)	0 (0)	0 (0)	0 (0)	2 (1.69)	0 (0)
1 AI, 1 IO	1 (0.37)	0 (0)	0 (0)	0 (0)	1 (0.85)	0 (0)
2 AI, 1 IO	1 (0.37)	0 (0)	0 (0)	0 (0)	1 (0.85)	0 (0)
2 AI, 3 IO	1 (0.37)	0 (0)	0 (0)	0 (0)	1 (0.85)	0 (0)
3 AI, 2 IO	1 (0.37)	0 (0)	0 (0)	0 (0)	1 (0.85)	0 (0)
2 AI, 1 OI	1 (0.37)	0 (0)	0 (0)	0 (0)	1 (0.85)	0 (0)
2 AI, 2 OI	1 (0.37)	0 (0)	0 (0)	0 (0)	1 (0.85)	0 (0)
1 IO	3 (1.12)	0 (0)	0 (0)	0 (0)	3 (2.54)	0 (0)
2 IO	5 (1.87)	0 (0)	0 (0)	0 (0)	5 (4.24)	0 (0)

AI: all-inside, IO: inside-out, OI: outside-in.

**Table 3:** Radial tears.

Variables	Both Menisci		Medial Meniscus		Lateral Meniscus	
	Number (%)	Failures (%)	Number (%)	Failures (%)	Number (%)	Failures (%)
No. of tears	12	0 (0)	3 (25)	0 (0)	9 (75)	0 (0)
Radial location						
Anterior horn	1 (8.33)		0 (0)		1 (100)	
Body/posterior horn	11 (91.67)		3 (27.27)		8 (72.73)	
Tear subtype						
Chahla II	8 (66.67)		3 (37.5)		5 (62.5)	
Chahla III	2 (16.67)		0 (0)		2 (100)	
Chahla IV	2 (16.67)		0 (0)		2 (100)	
Chahla V	0 (0)		0 (0)		0 (0)	
No. of repairs	31		4 (12.9)		27 (87.1)	
AI	24 (77.42)		4 (16.67)		20 (83.33)	
IO	7 (22.58)		0 (0)		7 (100)	
OI	0 (0)		0 (0)		0 (0)	
Technique(s) applied						
1 AI	2 (16.67)	0 (0)	2 (75.00)	0 (0)	0 (0)	0 (0)
2 AI	4 (33.33)	0 (0)	1 (25.00)	0 (0)	3 (33.33)	0 (0)
4 AI	1 (8.33)	0 (0)	0 (0)	0 (0)	1 (11.11)	0 (0)
5 AI	1 (8.33)	0 (0)	0 (0)	0 (0)	1 (11.11)	0 (0)
1 AI, 1 IO	1 (8.33)	0 (0)	0 (0)	0 (0)	1 (11.11)	0 (0)
1 AI, 2 IO	2 (16.67)	0 (0)	0 (0)	0 (0)	2 (22.22)	0 (0)
2 AI, 2 IO	1 (8.33)	0 (0)	0 (0)	0 (0)	1 (11.11)	0 (0)

AI: all-inside, IO: inside-out, OI: outside-in.

**Table 4:** Longitudinal lesions.

Variables	Both Menisci		Medial Meniscus		Lateral Meniscus	
	Number (%)	Failures (%)	Number (%)	Failures (%)	Number (%)	Failures (%)
No. of tears	80	1 (1.25)	39 (48.75)	0 (0)	41 (51.25)	1 (100)
Radial location						
Anterior horn	0 (0)		0 (0)		0 (0)	
Body/posterior horn	80 (100)		39 (48.75)		41 (51.25)	
No. of repairs	154		74 (48.05)		80 (51.95)	
AI	150 (97.4)		72 (48)		78 (52)	
IO	4 (2.6)		2 (50)		2 (50)	
OI	0 (0)		0 (0)		0 (0)	
Technique(s) applied						
1 AI	23 (28.75)	1 (100)	12 (30.77)	0 (0)	11 (26.83)	1 (100)
2 AI	40 (50)	0 (0)	20 (51.28)	0 (0)	20 (48.78)	0 (0)
3 AI	13 (16.25)	0 (0)	6 (15.38)	0 (0)	7 (17.07)	0 (0)
4 AI	1 (1.25)	0 (0)	0 (0)	0 (0)	1 (2.44)	0 (0)
1 AI, 1 IO	2 (2.5)	0 (0)	0 (0)	0 (0)	2 (4.88)	0 (0)
2 AI, 2 IO	1 (1.25)	0 (0)	1 (2.56)	0 (0)	0 (0)	0 (0)

AI: all-inside, IO: inside-out, OI: outside-in.

**Table 5:** Bucket-handle meniscal tears.

Variables	Both Menisci		Medial Meniscus		Lateral Meniscus	
	Number (%)	Failures (%)	Number (%)	Failures (%)	Number (%)	Failures (%)
No. of tears	55	4 (7.27)	34 (61.82)	3 (75)	21 (38.18)	1 (25)
Radial location						
Anterior horn	0 (0)		0 (0)		0 (0)	
Body/posterior horn	55 (100)		34 (61.82)		21 (38.18)	
No. of repairs	193		116 (60.10)		77 (39.90)	
AI	165 (85.49)		100 (60.61)		65 (39.39)	
IO	24 (12.44)		15 (62.50)		9 (37.50)	
OI	4 (2.07)		1 (25)		3 (75)	
Technique(s) applied						
1 AI	2 (3.46)	0 (0)	0 (0)	0 (0)	2 (9.52)	0 (0)
2 AI	11 (20.00)	0 (0)	8 (23.53)	0 (0)	3 (14.29)	0 (0)
3 AI	14 (25.45)	1 (25)	10 (29.41)	1 (33.33)	4 (19.05)	0 (0)
4 AI	9 (16.36)	1 (25)	5 (14.71)	1 (33.33)	4 (19.05)	0 (0)
5 AI	2 (3.46)	1 (25)	2 (5.88)	1 (33.33)	0 (0)	0 (0)
2 AI, 1 IO	1 (1.82)	0 (0)	1 (2.94)	0 (0)	0 (0)	0 (0)
2 AI, 2 IO	2 (3.46)	0 (0)	1 (2.94)	0 (0)	1 (4.76)	0 (0)
2 AI, 1 IO, 1 OI	1 (1.82)	0 (0)	1 (2.94)	0 (0)	0 (0)	0 (0)
3 AI, 1 IO	1 (1.82)	0 (0)	1 (2.94)	0 (0)	0 (0)	0 (0)
3 AI, 2 IO	6 (10.91)	0 (0)	5 (14.71)	0 (0)	1 (4.76)	0 (0)
3 AI, 1 IO, 1 OI	1 (1.82)	0 (0)	0 (0)	0 (0)	1 (4.76)	0 (0)
4 AI, 1 IO	3 (5.45)	0 (0)	0 (0)	0 (0)	3 (14.29)	0 (0)
4 AI, 2 OI	1 (1.82)	0 (0)	0 (0)	0 (0)	1 (4.76)	0 (0)
5 AI, 1 IO	1 (1.82)	1 (25)	0 (0)	0 (0)	1 (4.76)	1 (100)

AI: all-inside, IO: inside-out, OI: outside-in.

**Table 6:** Meniscal ramp lesions.

Variables	Medial Meniscus	
	Number (%)	Failures (%)
No. of tears	94	0 (0)
Radial location		
Anterior horn	0 (0)	
Body/posterior horn	94 (100)	
No. of repairs	140	
AI	140 (100)	
IO	0 (0)	
OI	0 (0)	
Technique applied		
1 AI	55 (58.51)	0 (0)
2 AI	32 (34.04)	0 (0)
3 AI	7 (7.45)	0 (0)

AI: all-inside, IO: inside-out, OI: outside-in.

**Table 7:** Meniscal root tears.

Variables	Both Menisci		Medial Meniscus		Lateral Meniscus	
	Number (%)	Failure (%)	Number (%)	Failure (%)	Number (%)	Failure (%)
No. of tears	61	0 (0)	8 (13.11)	0 (0)	53 (86.89)	0 (0)
Tear subtype						
LaPrade I	49 (80.33)		7 (14.29)		42 (85.71)	
LaPrade II–V	12 (19.67)		1 (8.33)		11 (91.67)	
No. of repairs	82		14 (17.07)		68 (82.93)	
AI	70 (85.37)		13 (18.57)		57 (81.42)	
PU	12 (14.63)		1 (8.33)		11 (91.67)	
Technique(s) applied						
1 AI	34 (55.74)	0 (0)	4 (50.00)	0 (0)	30 (56.60)	0 (0)
2 AI	13 (21.31)	0 (0)	2 (25.00)	0 (0)	11 (20.75)	0 (0)
3 AI	2 (3.28)	0 (0)	1 (12.50)	0 (0)	1 (1.89)	0 (0)
2 AI, 1 PU	2 (3.28)	0 (0)	1 (12.50)	0 (0)	1 (1.89)	0 (0)
PU	10 (16.39)	0 (0)	0 (0)	0 (0)	10 (18.87)	0 (0)

AI: all-inside, PU: pull-out.

**Table 8:** Complex meniscal tears.

Variables	Both Menisci		Medial Meniscus		Lateral Meniscus	
	Number (%)	Failures (%)	Number (%)	Failures (%)	Number (%)	Failures (%)
No. of tears	67	1 (1.49)	32 (47.76)	1 (100)	35 (52.24)	0 (0)
Radial location						
Anterior horn	0 (0)		0 (0)		0 (0)	
Body/posterior horn	67 (100)		32 (47.76)		35 (52.24)	
No. of repairs	159		65 (40.88)		94 (59.12)	
AI	146 (91.82)		63 (43.15)		83 (56.85)	
IO	9 (5.66)		2 (22.22)		7 (77.78)	
OI	4 (2.52)		0 (0)		4 (100)	
Technique(s) applied						
1 AI	17 (25.37)	0 (0)	13 (40.63)	0 (0)	4 (11.43)	0 (0)
2 AI	20 (29.85)	0 (0)	9 (28.13)	0 (0)	11 (31.43)	0 (0)
3 AI	16 (23.88)	1 (100)	6 (18.75)	1 (100)	10 (28.57)	0 (0)
3 AI, 1 IO	2 (2.99)	0 (0)	2 (6.25)	0 (0)	0 (0)	0 (0)
4 AI	3 (4.48)	0 (0)	2 (6.25)	0 (0)	1 (2.86)	0 (0)
5 AI	2 (2.99)	0 (0)	0 (0)	0 (0)	2 (5.71)	0 (0)
1 AI, 2 IO	1 (1.49)	0 (0)	0 (0)	0 (0)	1 (2.86)	0 (0)
2 AI, 1 IO	2 (2.99)	0 (0)	0 (0)	0 (0)	2 (5.71)	0 (0)
2 AI, 2 IO	1 (1.49)	0 (0)	0 (0)	0 (0)	1 (2.86)	0 (0)
3 AI, 1 IO	1 (1.49)	0 (0)	0 (0)	0 (0)	1 (2.86)	0 (0)
1 AI, 2 OI	1 (1.49)	0 (0)	0 (0)	0 (0)	1 (2.86)	0 (0)
2 AI, 2 OI	1 (1.49)	0 (0)	0 (0)	0 (0)	1 (2.86)	0 (0)

AI: all-inside, IO: inside-out, OI: outside-in.

## Data Availability

Data are available upon reasonable request.

## Abbreviations

The following abbreviations are used in this manuscript:

HCT	Horizontal cleavage tear
RT	Radial tear
MRL	Meniscal Ramp Lesion
MRT	Meniscal root tears
LT	Longitudinal tear
CMT	Complex Meniscal Tear
BHMT	Bucket-handle meniscal tear
MRI	Magnetic resonance imaging
ACL	Anterior cruciate ligament
MTL	Menisco-tibial ligament
MCL	Menisco-capsular ligament
SD	Standard deviation
AI	All-inside
IO	Inside-out
OI	Outside-in
PU	Pull-out
